# Time Delay Study of Ultrasonic Gas Flowmeters Based on VMD–Hilbert Spectrum and Cross-Correlation

**DOI:** 10.3390/s24051462

**Published:** 2024-02-23

**Authors:** Lingcai Kong, Liang Zhang, Hulin Guo, Ning Zhao, Xinhu Xu

**Affiliations:** 1 Thermometry Devision, National Institute of Metrology, Beijing 100029, China; k18838711291@163.com; 2 School of Quality and Technical Supervision, Hebei University, Baoding 071000, China; zhaoning1983@tju.edu.cn (N.Z.); xuxinhu@stumail.hbu.edu.cn (X.X.); 3 Zhengzhou Institute of Metrology, Zhengzhou 450001, China; guohulin@zim.ac.cn

**Keywords:** ultrasonic gas flowmeter, VMD, signal processing, time delay, Hilbert transform

## Abstract

The accuracy of ultrasonic flowmeter time delay measurement is directly affected by the processing method of the ultrasonic echo signal. This paper proposes a method for estimating the time delay of the ultrasonic gas flowmeter based on the Variational Mode Decomposition (VMD)–Hilbert Spectrum and Cross-Correlation (CC). The method improves the accuracy of the ultrasonic gas flowmeter by enhancing the quality of the echo signal. To denoise forward and reverse ultrasonic echo signals collected at various wind speeds, a Butterworth filter is initially used. The ultrasonic echo signals are then analyzed by Empirical Mode De-composition (EMD) and VMD analysis to obtain the Intrinsic Mode Function (IMF) containing distinct center frequencies, respectively. The Hilbert spectrum time–frequency diagram is used to evaluate the results of the VMD and EMD decompositions. It is found that the IMF decomposed by VMD has a better filtering performance and better anti-interference performance. Therefore, the IMF with a better effect is selected for signal reconstruction. The ultrasonic time delay is then calculated using the Cross-Correlation algorithm. The self-developed ultrasonic gas flowmeter was tested on the experimental platform of the gas flow standard devices using this signal processing method. The results show a maximum indication error of 0.84% within the flow range of 60–606 m^3^/h, with a repeatability of no more than 0.29%. These results meet the 1-level accuracy requirements as outlined in the national ultrasonic flowmeters calibration regulation JJG1030-2007.

## 1. Introduction

The ultrasonic gas flowmeter is a flow measurement instrument that utilizes the influence of ultrasonic waves on pipeline fluid [1]. It features the advantages of robust anti-interference capability, a wide measurement range, improved accuracy, and excellent stability [2,3,4]. Thus, it finds extensive application in several fields, including natural gas, petroleum, and aerospace [5,6].

The ultrasonic gas flowmeter utilizes three measurement techniques: the time difference method, Doppler effect method, and beam shift method [7]. The time difference method calculates flow rate by measuring the time of flight of forward and reverse ultrasonic signals in the fluid [8,9]. The Doppler effect method calculates scatterer flow velocity for measurement purposes by utilizing the Doppler shift phenomenon of acoustic waves. This is achieved through the detection of the Doppler shift generated by acoustic waves emitted from the source and received at the other end [10]. The method for measuring flow using an ultrasonic deflection angle is known as the beam deflection method, which is suitable for high-flow-rate scenarios [11]. Compared to the Doppler effect and beam deflection methods, the time difference method is less sensitive to particulates and is characterized by a broad measurement range, high reliability, and anti-interference, which contributes to its widespread use in ultrasonic gas flowmeters [12].

The ultrasonic flowmeter that uses the time difference method relies heavily on signal processing technology. There are three common signal processing approaches: the threshold method, the feature point method, and the Cross-Correlation method [13]. The threshold method determines the signal’s arrival time by setting one or more thresholds [14,15]. The feature point method determines the signal time of flight by fitting feature points based on the contours of echo signals and each peak point [16]. The Cross-Correlation method calculates the correlation function of the forward and reverse echo signals and determines the signal delay from its maximum [17]. The thresholding method is a simple and fast technique, but it is more susceptible to misjudgment [15,18]. The feature point method needs to be fitted according to the signal contour and peak points, and the feature parameters need to be calculated for each device, which involves a large amount of computation. In contrast, the Cross-Correlation method used in this study is applicable to cases where there is a similarity between forward and reverse signals. The computational method is mature and generalizable [7,19].

In flow measurements, experimental equipment, circuit systems, and other measurement environments introduce some degree of noise and baseline drift. It should be noted that these factors may impact the accuracy of the measurements [20]. Therefore, effectively filtering the noise and baseline drift in the ultrasonic echo signal is essential for improving the accuracy of the ultrasonic flowmeter.

Signal filtering methods for ultrasonic flowmeters comprise wavelet transform (WT), spectral peak analysis, empirical mode decomposition (EMD), and variational mode decomposition (VMD). WT provides a time–frequency window for signals to change with frequency, adapting to the requirements of time–frequency signal analysis. In 2020, Matthias Bächle et al. researched noise suppression techniques for signal processing of ultrasonic flowmeters using time difference methods. Their approach enhanced the signal-to-noise ratio through wavelet transform and adaptive filtering techniques [21]. It is important to note that the choice of wavelet basis function heavily affects the effectiveness of the wavelet transform. An unsuitable wavelet basis function may cause signal distortion, blurring, or inaccurate signal extraction. In 2016, Donghong Liu presented a spectral peak analysis-based signal processing technique [22]. However, using the spectral analysis method to directly filter uncorrelated signals results in a loss of signal energy. Ultrasonic signals can be adaptively decomposed using the EMD algorithm [23]. Tao Meng also presented a correlation analysis technique based on EMD, which separates overlapping fluctuations from various signal sources [24]. This method furnishes accurate insights into the detection and elimination of the originators of the fluctuations. However, the EMD method possesses a recursive property of modal decomposition, thus encountering end-point effects and modal aliasing when the modal components share similar frequencies. This limitation impedes the effective isolation of ultrasound echo signals from noise. On the other hand, VMD effectively mitigates modal mixing and endpoint effects caused by EMD. VMD is a technique for signal decomposition and modal analysis that can adapt to the desired signal and perform filtering model correction [25]. In a study by Sauvik Biswas et al., an intelligent fault detection and classification for UPFC transmission lines and wind farms was developed based on variational modal decomposition–CNN using VMD to decompose and extract optimal signals from locally measured current signals [26]. The VMD algorithm decomposes the original signal into multiple intrinsic modal functions (IMFs) with different center frequencies. This allows for the feature judgment and reconstruction of different frequency components in the signal [25,26].

Ultrasonic flowmeters are classified as either gas or liquid flowmeters, depending on the medium being measured. In an ultrasonic liquid flowmeter, signal propagation experiences less attenuation, making it easier to handle [27]. In contrast, ultrasonic gas flowmeters experience greater signal attenuation and are susceptible to noise and signal fluctuations. Therefore, the design of ultrasonic gas flowmeters needs to focus on anti-interference [28]. In this paper, we propose a combination of the VMD–Hilbert Spectrum and CC to calculate the time delay of ultrasonic echo signals for an ultrasonic gas flowmeter. The VMD decomposition optimization and reconstruction of the echo signal address problems such as interference from the gas flow of other frequencies, signal attenuation in the gas, and a low signal-to-noise ratio. The methodology can effectively eliminate noise from the echo signal, thereby improving the accuracy and reliability of the flow quantification achieved by ultrasonic flowmeters.

## 2. Theory

### 2.1. Ultrasonic Flowmeters Time Difference Method Principle

**Figure 1:**
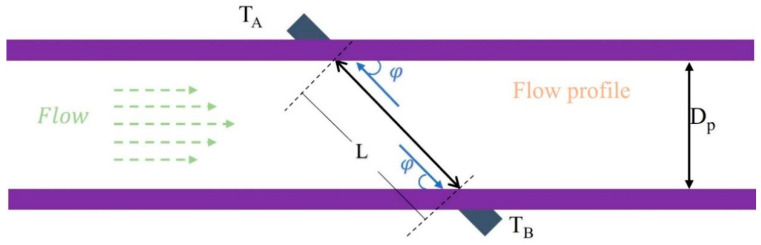
Schematic diagram illustrating the principle of the time difference method.

The time difference method for ultrasonic flowmeters is illustrated in Figure 1. The flow rate affects the speed of propagation of the ultrasonic waves in the fluid, so the time difference between the upstream and downstream ultrasonic travel can be measured to calculate the flow rate and volume.

The linear velocity V is obtained from Equation (1).

The liquid flows in a left-to-right direction. $T_{A}$ refers to a downstream ultrasonic transducer, and $T_{B}$ indicates a counterflow ultrasonic transducer. The angle between these transducers and the pipe is φ. The pipe diameter is $D_{p}$, the straight-line distance between the fronts of the two transducers is L, and the flow velocity through the pipe is V. The transmission speed of the acoustic wave in the gaseous medium is c.

(1) $$V=\frac{c^{2}(t_{2}-t_{1})}{2Lcos\varphi }=\frac{c^{2}∆t}{2Lcos\varphi }$$

where ∆t represents the time difference between the transmission of the ultrasonic signal in the acoustic channel with and against the flow. Correction factors vary depending on the mean surface flow rates of different fluids within a pipe. Let K be the correction factor and S be the cross-sectional area of the pipe. The pipe’s flow rate, Q, is determined via Equation (2).

(2) $$Q=KSV={K\pi (\frac{D_{p}}{2})}^{2}\frac{c^{2}∆t}{2Lcos\varphi }$$

The critical point of the problem is whether the time difference ∆t is accurately measured given the other parameters in Equation (2) are accurately measured.

### 2.2. The Principle of Cross-Correlation

**Figure 2:**
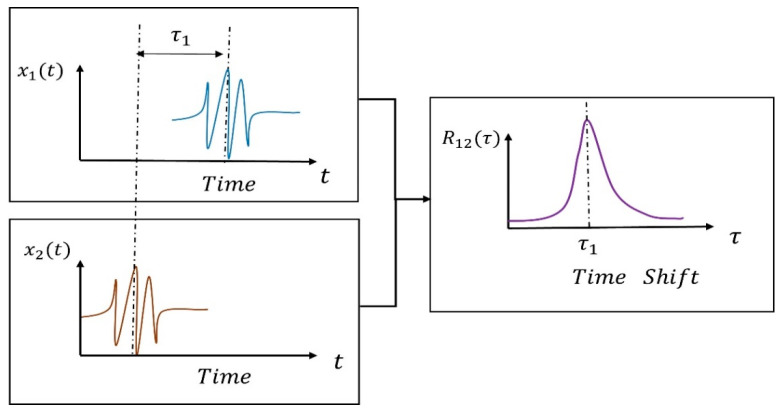
Schematic diagram of the principle of Cross-Correlation.

The Cross-Correlation principle is illustrated in Figure 2, and the delay outcomes can be obtained by computing the Cross-Correlation function between the echo signals of the cis-counterflow.

In this study, the received signals from two ultrasonic transducers are used to calculate the Cross-Correlation and obtain the time difference ∆t.

The ultrasonic transducer $T_{A}$ emits the signal s(t), and random white noise $n_{1}(t)$ is added during transmission of the acoustic channel to obtain $x_{1}(t)$ with accompanying noise. Likewise, the ultrasonic transducer $T_{B}$ emits the signal s(t), and random white noise $n_{2}(t)$ is added during transmission of the acoustic channel to obtain $x_{2}(t)$. The ratio of the amplitude of the received signals of $T_{A}$ and $T_{B}$ is represented by λ, while τ represents the time delay value of the two received signals.

(3) $$x_{1}\left(t\right)=s\left(t\right)+n_{1}(t)$$

(4) $$x_{2}\left(t\right)=\lambda s\left(t+\tau \right)+n_{2}(t)$$

Equations (3) and (4) are calculated in relation to one another to derive Equation (5), which represents the Cross-Correlation function.

(5) $$R_{12}(\tau )=\int_{-\frac{T}{2}}^{\frac{T}{2}}x_{1}\left(t\right)x_{2}\left(t+\tau \right)dt$$

When the time delay (τ) is equal to ∆t, the Cross-Correlation function can reach its maximum value, denoted as ${{[R}_{12}(\tau )]}_{max}$.

### 2.3. Variable Mode Decomposition (VMD) Principle

Variable Mode Decomposition (VMD) is a signal decomposition method that facilitates modal analysis while adapting to the intended signal and refining the filtering model [25]. The method was initially suggested by Dragomiretskiy et al. in 2014 [25]. The fundamental concept of VMD is to decompose the signal iteratively into a sequence of Intrinsic Mode Functions (IMFs), each displaying diverse frequency and bandwidth qualities. The modal component function is Equation (6):

(6) $$u_{k}\left(t\right)=A_{k}(t)\cos(\phi_{k}(t))$$

where $u_{k}\left(t\right)$, $A_{k}(t)$, and $\phi_{k}(t)$ denote the component signal, envelope amplitude, and instantaneous phase, respectively. The variational problem of decomposing modal components with various center frequencies and amplitudes can be addressed using the constrained variational model. The model described in Equation (7) is used to construct the model.

(7) $$\begin{array}{c} min\\ \left\{u_{k}\}\{\omega_{k}\right\} \end{array}\begin{array}{c} \left\{\sum_{k}\left||\;\partial_{t}\left[\left(\delta \left(t\right)+\frac{j}{\pi t}\right)u_{k}\left(t\right)\right]e^{-j\omega_{k}t}\;|\right|\begin{array}{c} 2\\ 2 \end{array}\right\}\\ s.t.\sum_{k}u_{k}=f \end{array}$$

Each modal component signal, $\left\{u_{k}\right\}$, has a center frequency of $\left\{\omega_{k}\right\}$. The impulse function is represented by $\delta \left(t\right)$, k signifies the number of decomposed modes, and $\partial_{t}$ refers to the Dirac distribution. Equally, $\sum_{k}$ is understood as the summation over all modes. The original signal is denoted by f. In Equation (8), the augmented Lagrangian function is utilized to transform the constrained variational problem into an unconstrained variational problem.

(8) $$\begin{array}{c} L\left(\left\{u_{k}\right\},\left\{\omega_{k}\right\},\lambda \right)=\alpha \sum_{k}∥\partial_{t}\left[\left(\delta \left(t\right)+\frac{j}{\pi t}\right)u_{k}\left(t\right)\right]e^{-j\omega_{k}t}\;∥\begin{array}{c} 2\\ 2 \end{array}\\ +∥f\left(t\right)-\sum_{k}u_{k}\left(t\right)∥\begin{array}{c} 2\\ 2 \end{array}+<\lambda \left(t\right),f\left(t\right)-\sum_{k}u_{k}\left(t\right)> \end{array}$$

In Equation (8), α represents the quadratic penalty factor, while λ(t) denotes the Lagrange operator. The components of $u_{k}$, $\omega_{k}$, and $\lambda_{k}$ can be obtained from the frequency domain utilizing the functions. The specific steps are listed below:

(a) Initialize $\left\{u_{k}^{1}\right\}$, $\left\{\omega_{k}^{1}\right\}$, $\left\{\lambda_{k}^{1}\right\}$ while setting n=0.
(b) n=n+1 iterating from 0.
(c) Equations (9)–(11) update ${\hat{u}}_{k}$, ${\hat{\omega }}_{k}$, and ${\hat{\lambda }}_{k}$, respectively.

(9) $${\hat{u}}_{k}^{n+1}\left(\omega \right)=\frac{\hat{f}\left(\omega \right)-\sum_{i\neq k}{\hat{u}}_{i}\left(\omega \right)+\frac{\hat{\lambda }(\omega )}{2}}{1+{2\alpha (\omega -\omega_{k})}^{2}}$$

(10) $${\hat{\omega }}_{k}^{n+1}\left(\omega \right)=\frac{\int_{0}^{\infty }\omega |{{\hat{u}}_{k}\left(\omega \right)|}^{2}d\omega }{\int_{0}^{\infty }|{{\hat{u}}_{k}\left(\omega \right)|}^{2}d\omega }$$

(11) $${\hat{\lambda }}_{k}^{n+1}\left(\omega \right)={\hat{\lambda }}_{k}^{n}\left(\omega \right)+o[\hat{f}\left(\omega \right)-\sum_{k=1}^{k}{\hat{u}}_{k}^{n+1}\left(\omega \right)]$$

${\hat{\omega }}_{k}^{n+1}\left(\omega \right)$ represents the power spectrum center of the corresponding modal component. The Fourier transforms of ${\hat{u}}_{k}^{n+1}$, $f\left(t\right)$, and $\lambda \left(t\right)$ correspond to ${\hat{u}}_{k}^{n+1}\left(\omega \right)$, $\hat{f}\left(\omega \right)$, and $\hat{\lambda }(\omega )$, respectively.

(d) Repeat steps (a) and (b) with continuously updated iterations until the end condition is satisfied.

(12) $$\frac{\sum_{k}∥{\hat{u}}_{k}^{n+1}-{\hat{u}}_{k}^{n}∥\begin{array}{c} 2\\ 2 \end{array}}{∥{\hat{u}}_{k}^{n}∥\begin{array}{c} 2\\ 2 \end{array}}<\epsilon \;,\;\epsilon =tolerance$$

In Equation (12), ϵ represents the degree of convergence accuracy.

**Figure 3:**
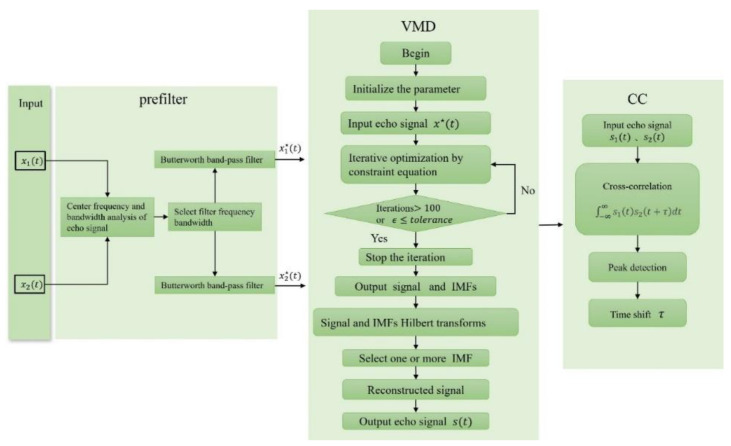
Workflow diagram for this study.

This paper’s workflow is depicted in Figure 3. Initially, the collected cis-countercurrent echo signals ($x_{1}\left(t\right)$ and $x_{2}\left(t\right)$) are stored on a computer (PC). Afterwards, the echo signal is pre-filtered with a Butterworth filter to eliminate significant interferences and background noise. Next, the pre-filtered signal undergoes decomposition and reconstruction by VMD to eliminate the smaller noise and baseline drift. Subsequently, the two reconstructed signals undergo Cross-Correlation to obtain the time difference between cis and countercurrent flows.

## 3. System Construction and Experimental Setup

### 3.1. System Construction

In this paper, a prototype ultrasonic gas flowmeter based on the VMD–Hilbert Spectrum and CC algorithm is fabricated. It is tested on a gas flow standard device built in the laboratory.

**Figure 4:**
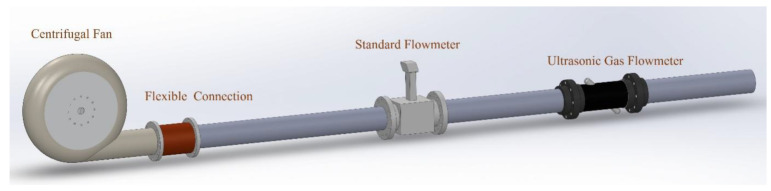
Schematic diagram of the gas flow standard device.

**Figure 5:**
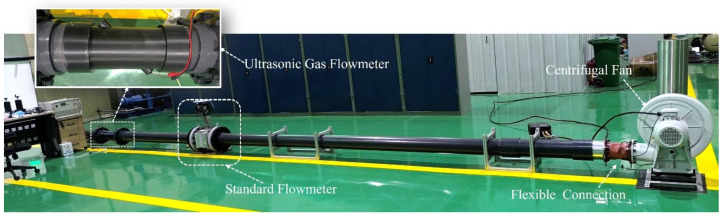
Physical drawing of the gas flow standard device.

Figure 4 shows the schematic diagram of the gas flow standard device. The device comprises a blower, two DN150 mm ultrasonic flowmeters, and multiple pipes. One of the blowers used is an Elektror centrifugal fan manufactured in Germany. The fan airflow is regulated using a frequency converter. The experiment utilizes two flowmeters, with one being the eight-channel ultrasonic gas flowmeter FLOW SICK600-XT from German manufacturer SICK, chosen as the standard flowmeter. The experimental standard flow rate is calibrated by using the measured results of this standard flowmeter, while the examined flowmeter in this experiment is a self-developed ultrasonic gas flowmeter prototype consisting of a PLA shell and a pair of ultrasonic sensors, with the angle between the sensors and the pipe being 45°. The sensors used in this study are the laboratory self-developed ultrasonic transducer, model K1-20 [29]. The center frequency of the sensor is 100 kHz. When the blower outlet, referenced standard meter, meter under inspection, and piping are assembled, each connection is ensured to be centered. The blower is positioned 30 diameters away from the inspected table. This ensures a steady flow of gas through both the standard and examined flowmeters. The physical drawing of the gas flow standard device used in the flow test is shown in Figure 5.

### 3.2. Experimental Verification

**Figure 6:**
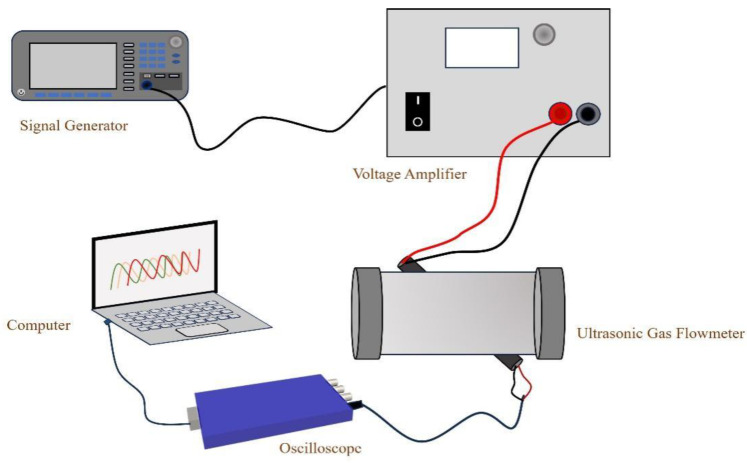
Echo Test System.

The echo test system is shown in Figure 6. The function generator produces 10 excitation pulse signals with an amplitude of 10 Vpp, a center frequency of 100 kHz, and a trigger period of 2 ms. The excitation pulse signal is then amplified to 200 Vpp by a power amplifier. This amplified signal excites the transducer at the transmitting end, generating ultrasonic waves that travel through the pipeline. The waves are then received by the receiving transducer and converted into electrical signals. The electrical signal converted by the transducer at the receiving end is collected by the oscilloscope and transmitted to the PC for VMD and CC processing.

During live-flow testing, the echo signal captured by the ultrasonic transducer at the receiving end can be contaminated with background noise. To enhance the signal quality and minimize the effect of noise on signal analysis, this study uses a Butterworth Bandpass Filter to preprocess the signal. The filter’s transition band features improve the filtering results’ smoothness and ability to retain the original signal characteristics. The filter parameters were configured based on the transducer employed in this investigation, which utilized a center frequency of 100 kHz and a ±10% bandwidth. After the signal has been pre-filtered, the filter’s transition bands enable improved smoothing of the filtered results while retaining the original signal’s characteristics. Nevertheless, the pre-filtered signal still carries low-energy noise and some degree of baseline drift.

To eliminate signal interference and isolate crucial data, two signal processing techniques, EMD and VMD, are employed in this study. These approaches effectively break down the original signal into various components, each characterized by unique amplitude and frequency features. In this study, a signal is considered valid if it has a center frequency of 100 kHz and a bandwidth of 10 kHz, which includes the necessary ultrasound echo signal. First, the EMD method decomposes the original signal into a sequence of intrinsic mode functions (IMFs) that represent the variations of the signal at varying frequencies and amplitudes. The time–frequency characteristics of these IMFs are analyzed to select one or several containing signals within the desired frequencies. Afterward, the valid signals are reconstructed by the echo signals. In this paper, the original signal also undergoes signal processing via the VMD method. This technique decomposes the signal into multiple Intrinsic Mode Functions (IMFs), each with unique frequency characteristics that provide information about the signal in distinct frequency bands. By performing a time–frequency analysis of the decomposed VMD modal functions, one or more may be selected as valid signals for reconstructing the echo signal. This signal effectively eliminates unwanted background interference noise and eliminates baseline drift caused by low-frequency signals and noise.

A realistic flow trial was conducted on the gas flow standard device using air as the medium. At the start of the experiment, we adjusted the frequency converter to stabilize the blower air volume at the flow measurement point. To ensure uniform pressure and temperature between the standard flowmeter and the examined flowmeter, we adjusted the frequency converter at the start of the experiment to stabilize the blower air volume at the flow measurement point. Once the values of the standard flowmeter and examined flowmeter have stabilized, we recorded the measurement results separately.

The process for verifying real-flow tests is as follows: Fifty echo signal sets are collected at a consistent flow rate. The VMD–Hilbert Spectrum method is utilized for signal decomposition, reconstruction, and additional processing, followed by CC algorithm processing to determine the time delay ∆t. Based on Equation (5) in this paper, the instantaneous flow rate $q_{i}$ can be calculated. The average flow rate Q is then determined by taking the arithmetic mean of 50 groups of instantaneous flow rates. The experiment was repeated 10 times under the same conditions. Ultimately, the average flow rate was determined using a homemade ultrasonic flowmeter and compared to the standard flow rate provided by FLOWSIC600-XT for conclusion comparison.

## 4. Results and Discussion

### 4.1. Signal Pre-Processing

**Figure 7:**
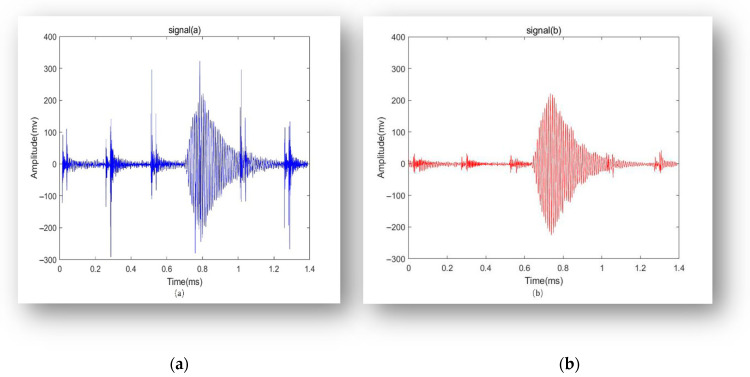
Echo Signal: (**a**) Before Filtering; (**b**) After filtering.

The comparison before and after filtering is shown in Figure 7. Compared to the original signal, the quality of the preprocessed signal has significantly improved. The signal-to-noise ratio is enhanced, and the reduction of noise and interference components has led to a clearer and more reliable signal.

**Figure 8:**
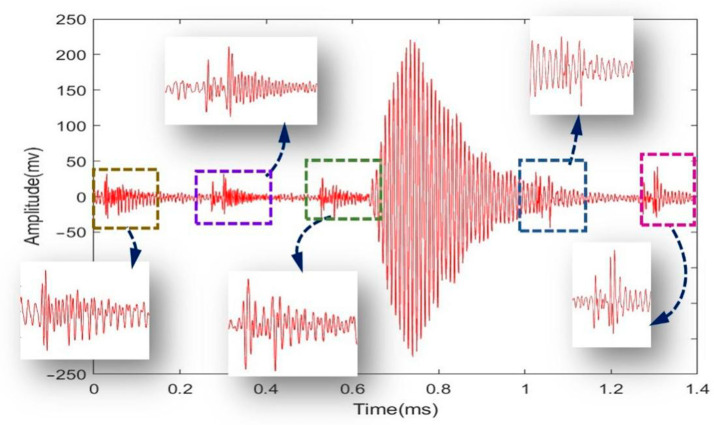
Detailed view of the echo signal after preprocessing.

Examining the preprocessed signal as shown in Figure 8, it is apparent that there is noise and baseline drift present within the signal at regular intervals. Both the EMD and VMD methods discussed in the following section will remove this noise and baseline drift.

### 4.2. Echo Signal Decomposition for EMD–Hilbert Spectrum

**Figure 9:**
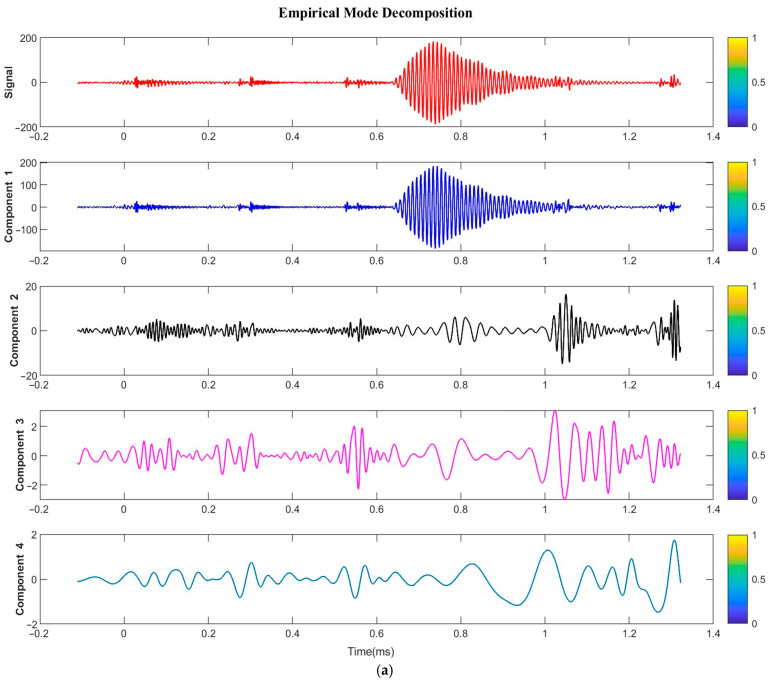

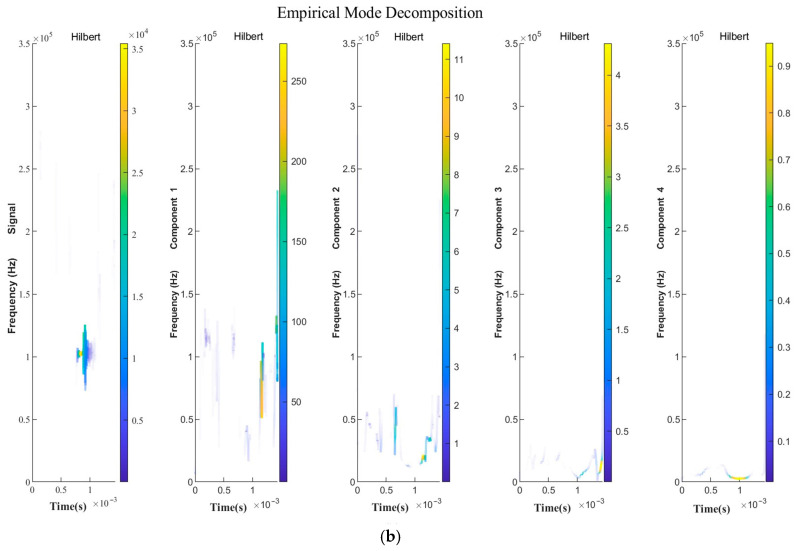
Signal and EMD components: (**a**) Time–domain plot; (**b**) Time–frequency spectrum.

The effective signal bandwidth in this study is 100 kHz ± 10%, as defined by the echo signal bandwidth. Figure 9 shows the imported original echo signal (“Signal”) and the four IMF components of the EMD decomposition (“Components 1–4”). Component 1 is identified as the required echo signal for this study, but it still contains noise at intervals. The time–frequency plot from the Hilbert transform in Figure 9b indicates that the Component 1 signal has a frequency range of 70–270 kHz, which is far beyond the bandwidth of the effective signal. The results demonstrate that the filtering and reconstruction process using EMD only removes a small portion of the noise, leaving some low-energy noise that affects the accuracy of the subsequent Cross-Correlation calculation.

### 4.3. Echo Signal Decomposition and Reconstruction for VMD–Hilbert Spectrum

**Figure 10:**
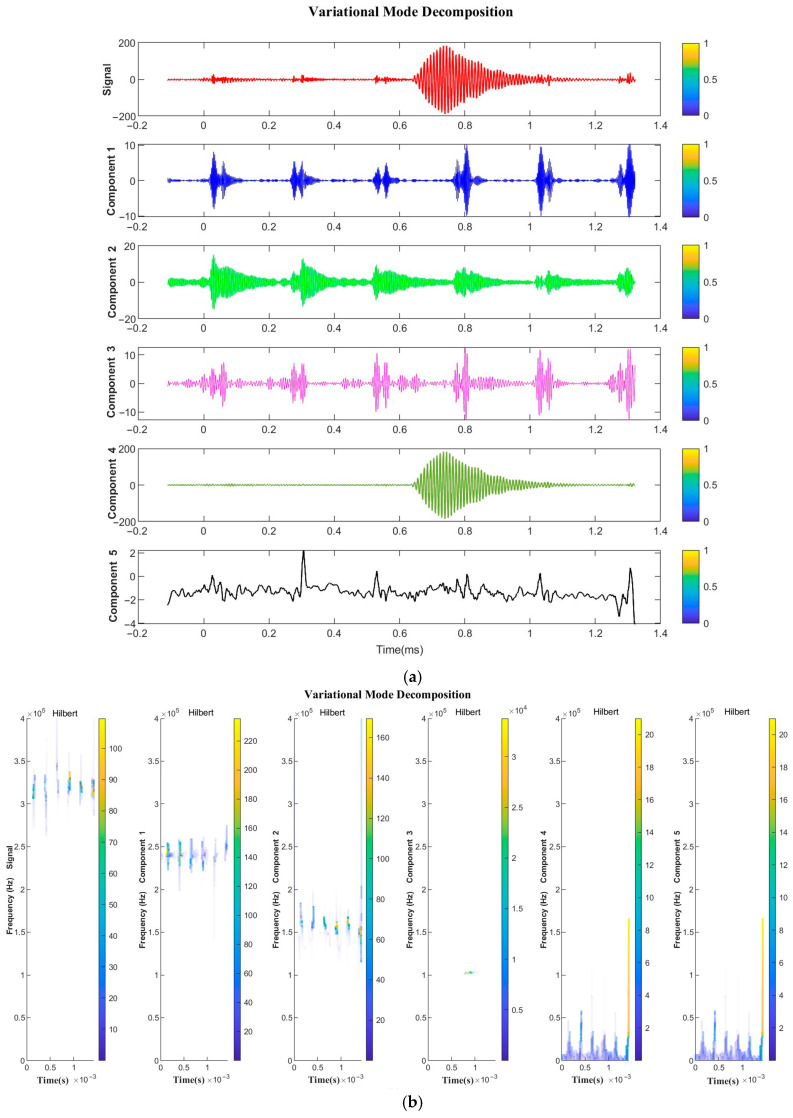
Signal and VMD components: (**a**) Time–domain plot; (**b**) Time–frequency spectrum.

Figure 10 shows the original imported echo signal labeled as “Signal” and the five IMFs decomposed by VMD labeled as “Components 1–5”. From Figure 10a, it can be clearly seen that Component 4 contains the echo signal required for this study, which is almost free of noise effects. The Hilbert transform time–frequency diagram in Figure 10b shows that Component 4 contains a frequency range of 108–110 kHz, which is the effective signal defined in this study and is near the center frequency of the ultrasonic transducer. The time–frequency analysis of Component 4 indicates that there are no signals in other frequency ranges except for the effective signal bandwidth range. The Component 4 signal obtained is relatively smooth and clean, with almost complete filtering of background noise. The results show that VMD has the following advantages over EMD:

(1) Precise decomposition ability is superior—VMD demonstrates high accuracy in signal decomposition and effectively breaks down complex signals into a sequence of modal functions with precise frequency bands and distinct physical significance. As compared to EMD, VMD exhibits greater stability in the signal decomposition process and maintains superior accuracy in decomposition.
(2) Higher anti-interference performance—VMD utilizes adaptive signal decomposition to extract noise-resistant signals, which outperforms EMD. The technique effectively eliminates the noise component, resulting in a smoother and cleaner signal.
(3) Greater frequency concentration—When decomposing a signal, VMD allows for the concentration of components in a specific frequency range of interest within certain modal functions. This method is superior to EMD, as it accurately extracts the frequency characteristics of the desired signal while excluding noise interference.
(4) Better adaptability—VMD is able to adapt and select the optimal decomposition scale based on signal characteristics. This capability enables VMD to better analyze signals with varied characteristics, leading to improved accuracy and robustness in signal decomposition.
(5) More efficient algorithms—Compared with traditional EMD methods, VMD is significantly more computationally efficient and faster. As a result, VMD has become widely adopted in fields that demand efficient processing, such as real-time and big data processing.

**Figure 11:**
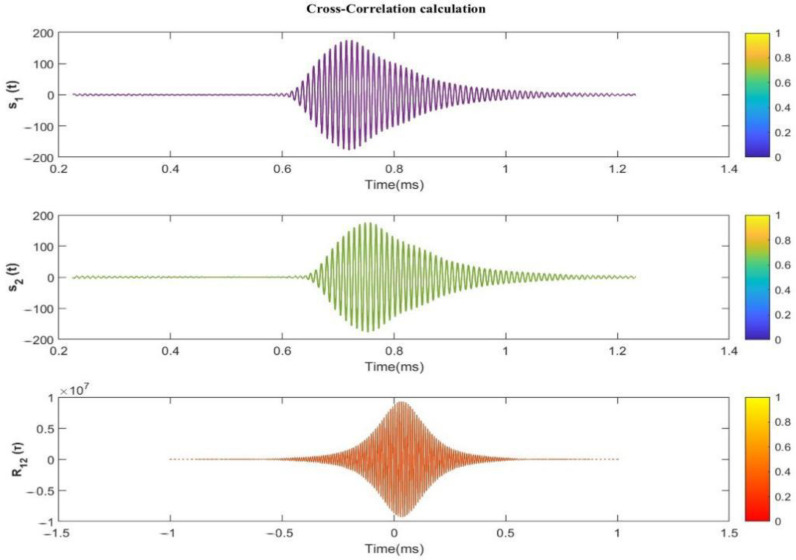
$s_{1}\left(t\right)$ and $s_{2}\left(t\right)$ Cross-Correlation waveforms.

Based on the results and analysis presented above, we selected Component 4 within VMD for further study. Using Component 4, we reconstructed the cis-countercurrent echo signal and obtained signals $s_{1}\left(t\right)$ and $s_{2}\left(t\right)$. The $s_{1}\left(t\right)$ and $s_{2}\left(t\right)$ signals undergo Cross-Correlation to yield the mutual correlation waveform, as demonstrated in Figure 11. By detecting the peak, the time delay result ∆t can be obtained from the $R_{12}(\tau )$ Cross-Correlation waveform.

According to the national testing regulations for ultrasonic flowmeters in China (JJG 1030-2007), 1-level precision ultrasonic gas flowmeters must adhere to value error and repeatability requirements outlined in Table 1. The value of $q_{t}$ represents the middle node of the low-flow and high-flow ranges. For flowmeters operating within the $q_{t}\leq q\leq q_{max}$ flow range, the maximum allowable error is ±1%. In the $q_{min}\leq q\leq q_{t}$ flow range, the maximum allowable error cannot exceed the maximum allowable error specified in Table 1 by more than two times. Moreover, for gas flowmeters, the $q_{t}$ corresponding flow rate must not exceed 3 m/s.

Table 2 displays the outcomes without conducting VMD and Hilbert Transform analysis and processing.

Table 3 displays the outcomes with processing and analysis via VMD and Hilbert Transform.

The experiments on actual flow conducted using the aforementioned signal processing algorithm encompass the range of flow between $60\;m^{3}/h$ and ${606\;m}^{3}/h$. When comparing Table 2 and Table 3, it can be observed that the relative error of the $q_{min}$ point in the small flow interval drops from 8.56% before data filtering to 0.84% after data filtering. Likewise, the relative error of the $q_{max}$ in the large flow interval also decreases from 3.51% before data filtering to 0.22% after data filtering. Meanwhile, within the flow range of $q_{min}\leq q<q_{t}$, the measurement error is below ±2% with a repeatability below 0.3%. Within the flow range of $q_{t}\leq q\leq q_{max}$, the measurement error is below ±1% with a repeatability below 0.2%. According to the experimental results, the application of the filtering algorithm results in a signal that is more accurate, more robust, and simultaneously conforms to the requirements of 1-level accuracy according to the national calibration regulations for ultrasonic flowmeters.

## 5. Conclusions

To enhance the accuracy of ultrasonic gas flowmeter measurements, this study presents a time delay estimation approach for ultrasonic gas flowmeters that utilizes the VMD–Hilbert spectrum and Cross-Correlation. This paper provides a detailed description of the interference experienced by the ultrasonic echo signal during real flow and the consequent signal processing procedure. Furthermore, the efficacy of the echo signal filtering algorithm is verified through the real-flow test. Analysis reveals significant reductions in relative measurement errors for low- and high-flow rates, decreasing from 8.56% to 0.84% and 3.51% to 0.22%, respectively, following signal filtration. The system measurements were found to be within the acceptable error limits of ±1% for the flow range of 61–606 m^3^/h, which is in line with the measurement error standards for class 1 instruments. After processing the data, the ultrasonic flowmeter exhibits improved robustness and measurement accuracy, resulting in significantly enhanced overall measurement performance. This paper’s research method has limitations. When the flow rate exceeds ${606\;m}^{3}/h$, the signal quality and amplitude decrease. This phenomenon may result from the vibration of the PVC pipe on the ultrasonic gas flowmeter measuring platform under high wind speeds or the increase in turbulence intensity in the pipe. Further testing and research will follow.

## Figures and Tables

**Table 1:** Error and Repeatability Requirements.

$\mathrm{Flow}\;\mathrm{Rate}\;(m^{3}/h)$	Mean Measurement Error (%)	Repeatability (%)
$q_{min}\leq q\leq q_{t}$	±2%	±0.4%
$q_{t}\leq q\leq q_{max}$	±1%	±0.2%

**Table 2:** Results without VMD–Hilbert Spectrum Processing.

Flow Rate Point	$\mathrm{Flow}\;\mathrm{Rate}\;(m^{3}/h)$	Measured Flow Rate (m^3^/h)	Mean Measurement Error (%)	Repeatability (%)
$q_{min}$	60.60	65.79	8.56	3.42
$1.5q_{min}$	90.91	96.64	6.30	2.71
$q_{t}$	121.20	127.71	5.37	2.46
$0.4q_{max}$	242.41	254.24	4.88	2.13
$0.7q_{max}$	424.20	440.84	3.92	2.01
$q_{max}$	606.01	627.29	3.51	1.94

**Table 3:** Results after VMD–Hilbert Spectrum Processing.

Flow Rate Point	$\mathrm{Flow}\;\mathrm{Rate}\;(m^{3}/h)$	$\mathrm{Measured}\;\mathrm{Flow}\;\mathrm{Rate}\;(m^{3}/h)$	Mean Measurement Error (%)	Repeatability (%)
$q_{min}$	60.60	61.11	0.84	0.29
${1.5q}_{min}$	90.91	91.58	0.73	0.23
$q_{t}$	121.20	121.75	0.45	0.19
$0.4q_{max}$	242.41	241.59	−0.34	0.16
$0.7q_{max}$	424.20	423.17	−0.24	0.12
$q_{max}$	606.01	607.34	0.22	0.09

## Data Availability

Data are contained within the article.
